# Draft genome sequence of *Mycobacterium* sp. SA01 isolated from *Sporobolus alterniflorus* seedlings collected in Cape Cod (USA)

**DOI:** 10.1128/mra.01025-24

**Published:** 2025-01-10

**Authors:** Elena L. Peredo, Suzanne Thomas, Zoe G. Cardon

**Affiliations:** 1 Thomas H. Gosnell School of Life Sciences, College of Science, Rochester Institute of Technology, Rochester, New York, USA; 2 The Ecosystems Center, Marine Biological Laboratory, Woods Hole, Massachusetts, USA; The University of Arizona, Tucson, Arizona, USA

**Keywords:** *Spartina alterniflora*, salt marsh, polycyclic aromatic hydrocarbons, *Mycobacterium*

## Abstract

A draft genome was generated for a strain of *Mycobacterium* closely related to *Mycobacterium* sp. ENV421 isolated *in vitro* from plants of smooth cordgrass germinated *in vitro* from seeds collected in a salt marsh in Cape Cod (USA). Genomic DNA was sequenced using paired-end Illumina technologies. Here, we report the classification and genome statistics of *Mycobacterium* sp. SA01. A strain of *Mycobacterium* was isolated from seedlings of *Sporobolus alterniflorus* germinated under in vitro conditions after surface sterilization. The seeds were collected in Little Swippewisett Marsh, Barnstable, MA, USA.

## ANNOUNCEMENT

*Mycobacterium* spp. are a diverse group of Gram-positive, rod-shaped bacteria belonging to the class Actinomycetia. Nontuberculous mycobacteria are typically nonpathogenic and found in many natural environments (1), including soils in bogs, forests, croplands, or livestock farms (2). *Mycobacterium* spp. are often capable of degrading polycyclic aromatic hydrocarbon compounds (PAHs) (3). PAHs are toxic molecules of natural or anthropogenic origin (4). Their presence is of special concern in coastal environments because marshes act as carbon sinks sequestering toxins from the atmosphere and water (5) and retaining petrol-derived PAH after oil spills. *Mycobacterium* spp. are frequently found in the rhizosphere, and it has been proposed that their presence in plant roots might enhance PAH degradation by facilitating the access to oxygen (6). Understanding the dynamics of PAH degradation by plant–microbe interactions is important for evaluating the overall health of coastal areas. Here, we report a draft genome of *Mycobacterium* sp. SA01, which was found in close association with seeds of *Sporobolus alterniflorus*.

Seeds of *Sporobolus alterniflorus* were collected in September–November 2023 in a salt marsh (Little Swippewisett, Massachusetts, USA, 41.576093 N 70.640647 W). Seeds were surface-sterilized using 20% bleach for 20 minutes and germinated in modified MS media (reference M527, Phytotech Labs, USA) supplemented with 3% sucrose. Seeds were cultivated at 20–22°C in an illuminated culture chamber (100 μE). A single type of bacteria, notably bright yellow, was observed growing around the roots of some of the vitroplants, which are plants grown in lab settings using sterile conditions and nutrient media. The bacterium was isolated by re-streaking in Luria–Bertani (LB) media. Samples were cultivated under the same conditions as the vitroplants. After three rounds of isolation in solid media, a colony was picked and used for DNA extraction using a Maxwell RSC system (AS1600, Promega, USA) according to the manufacturer’s instructions.

DNA libraries were prepared and sequenced by The Keck Facility at the Marine Biological Laboratory (Massachusetts, USA) using the Nextseq 500 platform (Illumina). This effort generated 9,441,397 paired-end reads (2 × 150 nt), which were trimmed to remove low-quality base calls and adapters using Trimmomatic v0.36 (7). A total of 96.6% of reads were retained. Their quality was assessed using FastQC v0.11.9 (8). Reads were assembled into contigs with Megahit v1.2.9 (9) using a minimum length of 2,000 bp. We used multiple approaches to ensure the absence of contaminants in the final genome assembly. First, we examined the GC content/coverage relationship and contig coverage, and then we employed dedicated tools to bin the assembly: MaxBin2 v2.2.4 (10), MetaBAT2 v1.7 (11), and CONCOCT v1.1 (12). All binning programs consistently identified the contigs as belonging to a single bin, representing a single genome.

The total genome length was determined as 5.9 Mb (5,900,438 bp) in 22 contigs with an N50 of 501 Kb and a GC content of 66.8%. CheckM v1.0.18 (13) assessed the completeness at 98.5% with <1% contamination. The genome was identified as 98.01% match to *Mycobacterium* sp. ENV421 (GCA_002887815.1) using average nucleotide identity (ANI) against publicly available reference genomes with FASTANI v0.1.3 (14) and through GTDB-Tk v2.3.2 (15). Genome annotation was performed with Prokaryotic Genome Annotation Pipeline (PGAP) v6.7 (16). A total of 5,695 genes were identified, of which 5,597 were protein-coding genes. Unless otherwise noted, all programs were run using default settings.

## Data Availability

The draft genome has been deposited in GenBank under the accession number JBGGNO000000000. The raw sequence file is available under the accession number SRR30003677. The BioProject can be found under the reference PRJNA1140187. The 16S ribosomal sequence is available at PQ094705.
